# Valproate Induced Hypertensive Urgency

**DOI:** 10.1155/2016/1458548

**Published:** 2016-06-15

**Authors:** Mauran Sivananthan, Sarah Mohiuddin

**Affiliations:** Child & Adolescent Psychiatry, University of Michigan, 4250 Plymouth Road, Ann Arbor, MI 48103, USA

## Abstract

Valproate is a medication used in the treatment of seizures, bipolar disorder, migraines, and behavioral problems. Here we present a case of an 8-year-old boy who presented with hypertensive urgency after initiation of valproate. Primary treatment of his hypertension was ineffective. Blood pressure stabilization was achieved following discontinuation of valproate. Clinicians should be aware of the risk of developing hypertensive urgency with administration of valproate.

## 1. Introduction

Valproate is a medication that is commonly used to treat bipolar disorder as well as multiple types of epilepsy [1]. It is also commonly used off-label for a variety of other psychiatric disorders. Valproate is associated with a number of side effects [2], but valproate induced hypertensive urgency has not been previously described.

## 2. Case

 A. R. was an 8-year-old white male with history of attention deficit hyperactive disorder (ADHD), oppositional defiant disorder (ODD), Fetal Alcohol Syndrome, and Wolff-Parkinson-White s/p ablation. He had previously been maintained on olanzapine for 1-2 months and guanfacine for 5 months. He was then hospitalized on a psychiatric inpatient unit for physical aggression and was medically cleared with stable vitals prior to admission. Once hospitalized, he was started on Depakote 125 mg twice daily with the first dose given in the evening of his admission and the second dose given the following morning.

Within hours of his second dose of valproate, he complained of headache and was found to have elevated blood pressure (BP) and rapid heart rate (HR). He was sent to a nearby emergency department (ED) where his vitals were stabilized, and he was sent back to inpatient psychiatry. The next day, he woke up with return of his frontal headache with BP again noted to be as high as 160/120 with HR between 120 and 140 and again was sent to the ED.

In the ED, valproate was discontinued and cardiac workup was negative. He was then admitted to nephrology for workup of secondary causes of hypertension. During his hospitalization, labs were within normal limits and renal ultrasound did not reveal abnormal pathology. On the 2nd day of hospitalization, recommendation was made to also discontinue olanzapine. He was discharged the following day with stable blood pressure ranging from 106 to 119/55 to 62 and a negative workup.

## 3. Discussion

This case describes a preadolescent patient who had valproate induced hypertensive urgency. Valproate is known to have a broad mechanism of action including blocking sodium channels, increasing GABA, and inhibiting glutamate/NMDA receptor mediated neuronal excitation [3].

Valproate has FDA approval for acute mania/mixed episode in bipolar disorder. Other indications for use include complex partial seizures, absence seizures, and migraine headache prophylaxis [1]. For many years, valproate has also been used off-label for a variety of severe psychiatric disorders such as schizophrenia, ODD, depression, and borderline personality disorder [4].

There are many known side effects of valproate including thrombocytopenia and hepatotoxicity [1]. Cardiovascular side effects are reported in 1–5% of patients taking valproate and include hypertension, tachycardia, and palpitations [2], though hypertensive urgency has not been previously described.

Hypertensive urgency is defined as an acute elevation in BP without severe, life-threatening symptoms or evidence of acute target organ damage [5]. Psychiatric medications that have previously been linked to hypertension include amphetamines and antidepressants such as venlafaxine, TCAs, and MAOIs [6].

Though there has not been a prior reported association between valproate and hypertensive urgency, it has been shown to cause hypertension [2]. Studies have linked the significant effects guanfacine can have on valproate metabolism [7] and the addition of valproate to an already high dose (5 mg total daily dose) of guanfacine that likely led to a rapid increase in valproate levels. Another possible mechanism could be explained by a shared mechanism of action of valproate with carbamazepine, which has been implicated in inducing hypertension [8].

Limitations of this case study include a potential delayed onset of olanzapine induced hypertensive urgency, although this has not been reported in the literature. There have been case reports of clozapine induced hypertension [9], and given similar structural similarities of clozapine to olanzapine, it is a possibility, though highly unlikely.

This case provides evidence for clinicians to monitor hypertensive urgency in patients treated with valproate, particularly those who are on multiple psychotropic medications.
